# Temperature-Controlled Asymmetric Transmission of Electromagnetic Waves

**DOI:** 10.1038/s41598-019-40791-4

**Published:** 2019-03-11

**Authors:** Meng Liu, Quan Xu, Xieyu Chen, Eric Plum, Hua Li, Xueqian Zhang, Caihong Zhang, Chongwen Zou, Jiaguang Han, Weili Zhang

**Affiliations:** 1 Center for Terahertz waves and College of Precision Instrument and Optoelectronics Engineering, Tianjin University and the Key Laboratory of Optoelectronics Information and Technology (Ministry of Education), Tianjin, 300072 China; 2 Centre for Photonic Metamaterials & Optoelectronics Research Centre, Zepler Institute, University of Southampton, Southampton, SO17 1BJ UK; 3 Research Institute of Superconductor Electronics, Nanjing University, Nanjing, 210093 China; 4 National Synchrotron Radiation Laboratory, University of Science and Technology of China, Hefei, 230029 China; 5 School of Electrical and Computer Engineering, Oklahoma State University, Stillwater, Oklahoma 74078 USA

## Abstract

Chiral materials can exhibit different levels of transmission for opposite propagation directions of the same electromagnetic wave. Here we demonstrate thermal switching of asymmetric transmission of linearly polarized terahertz waves. The effect is observed in a terahertz metamaterial containing 3D-chiral metallic inclusions and achiral vanadium dioxide inclusions. The chiral structure exhibits pronounced asymmetric transmission at room temperature when vanadium dioxide is in its insulator phase. As the metamaterial is heated, the insulator-to-metal phase transition of vanadium dioxide effectively renders the structure achiral and the transmission asymmetry vanishes. We demonstrate the effect numerically and experimentally, describe it analytically and explain the underlying physical mechanism based on simulated surface current distributions. Potential applications include directionally asymmetric active devices as well as intensity and polarization modulators for electromagnetic waves.

## Introduction

Polarization is a fundamental property of electromagnetic waves and the ability to manipulate polarization states underpins numerous applications throughout the electromagnetic spectrum. Established methods for polarization manipulation employ linear anisotropy, optical activity and the Faraday effect in crystals, which require long interaction lengths (compared to the wavelength) to accumulate significant polarization changes^1^. Linear anisotropy results from preferred directions in the material’s structure and manifests itself as different phase delays (linear birefringence, used in wave plates) and different transmission levels (linear dichroism, used in linear polarizers) for orthogonal, linear eigenpolarizations. Optical activity occurs in 3D-chiral media, i.e. media that are different from their mirror image, and manifests itself as different phase delays (circular birefringence, used in polarization rotators) and different transmission levels (circular dichroism, used in circular polarizers) for left-handed and right-handed circular eigenpolarizations. Linear anisotropy and optical activity are the same for opposite wave propagation directions. Directional asymmetries arise from the broken reciprocity of the wave-matter interaction under static magnetic field, which is known as the Faraday effect. The Faraday effect is characterized by interchanged phase delays and interchanged transmission levels for opposite propagation directions of its circularly polarized eigenstates (used in optical isolators and circulators)^2^. Metamaterials, which derive enhanced or novel electromagnetic properties from artificial structuring on the sub-wavelength scale, have enabled orders-of-magnitude enhancements of linear anisotropy^3–6^ and optical activity^7–12^ from microwave to optical frequencies, and led to the discovery of directionally asymmetric effects that are reciprocal. Directionally asymmetric transmission of circularly polarized waves occurs in lossy, anisotropic structures with 2D chirality (the 2D twist of spirals) and arises from different conversion efficiencies between left- and right-handed circularly polarized waves that interchange for opposite propagation directions (circular conversion dichroism)^13–16^. In contrast, directionally asymmetric transmission of linearly polarized waves occurs in anisotropic structures with 3D chirality (the 3D twist of helices) and arises from different conversion efficiencies between orthogonal linear polarizations that interchange for opposite propagation directions (linear conversion dichroism)^17–22^. Full control over the polarization of electromagnetic waves requires dynamic control over these polarization effects. Linear anisotropy and optical activity of liquid crystals are routinely controlled by electric field and various approaches to controlling these effects in metamaterials have been reported, for example, based on mechanical actuation^23,24^, photoexcitation of carriers in semiconductors^25–28^, coherent control of light-matter interactions^29^ and phase transitions^30–33^. The Faraday effect is easily controlled by magnetic field.

**Figure 1:**
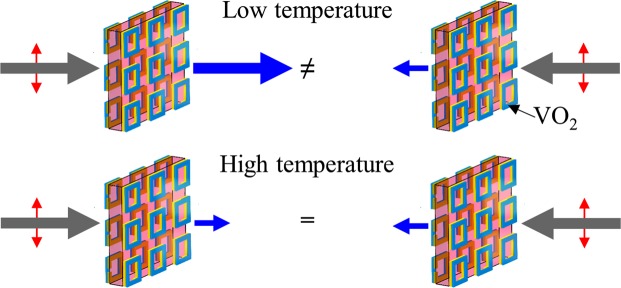
Temperature-controlled asymmetric transmission. The metamaterial transmits the same linearly polarized electromagnetic waves (gray arrows) asymmetrically at low temperatures (top) and symmetrically at high temperatures (bottom).

**Figure 2:**
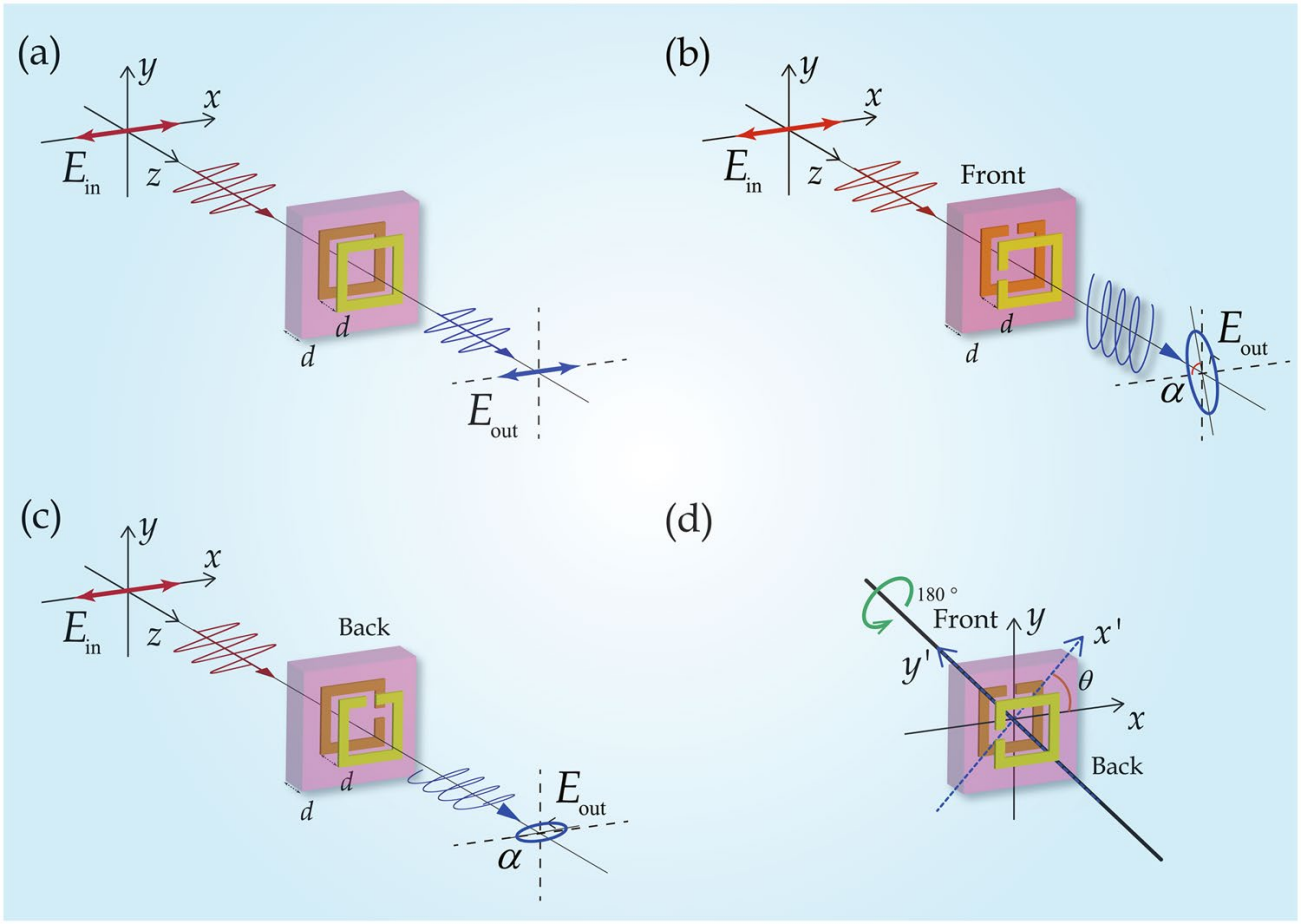
Metamaterial unit cells exhibiting symmetric and asymmetric transmission. (**a**) A unit cell with two identical metallic square rings on both sides of a dielectric spacer, where the two square rings are aligned along the *z* direction, transmits electromagnetic waves symmetrically. (**b**,**c**) Illumination of (**b**) front and (**c**) back of the unit cell for the proposed asymmetric transmission metamaterial composed of two 90°-twisted structurally identical SRRs separated by a dielectric spacer^19,20^. Illumination of the back of the sample is achieved by rotating the sample by 180° around the *y* axis. (**d**) Illustration of the asymmetric transmission metamaterial’s axis of twofold rotational symmetry *y'*.

Here we demonstrate dynamic control of directionally asymmetric transmission (Fig. 1). Using the insulator-to-metal phase transition of vanadium dioxide (VO_2_)^34,35^, we effectively switch between different metamaterial symmetries, resulting in thermal switching of directionally asymmetric transmission of linearly polarized terahertz waves. The effect is observed in a metamaterial structure based on anisotropic, 3D-chiral pairs of metallic split-ring-resonators (SRRs) overlapping with pairs of isotropic, achiral vanadium dioxide rings on opposite sides of a polyimide spacer (Fig. 2(b,c)). The first-order structural phase transition of VO_2_ from an insulating monoclinic phase to a metallic rutile phase^35^ effectively renders the structure achiral and isotropic by short-circuiting the split rings, resulting in temperature-control of asymmetric transmission accompanied by polarization modulation. Our approach may be applied to realize active polarization control devices and active asymmetric transmission devices of subwavelength thickness.

## Concepts and Theoretical Analysis

In order to design metamaterials with controllable asymmetric transmission for linearly polarized waves at normal incidence, we analyze the material symmetries that lead to presence and absence of the effect. The polarization properties of a slab can be described by a frequency-dependent Jones matrix (*T*_*ij*_), which links the complex electric field amplitudes of the incident wave's polarization components (*I*_*j*_) to those of the transmitted wave (*T*_*i*_) as shown in Eq. (1). For wave propagation onto the front of the structure, this takes the form

1 $$(\begin{array}{c}{T}_{x}\\ {T}_{y}\end{array})=(\begin{array}{cc}{T}_{xx} & {T}_{xy}\\ {T}_{yx} & {T}_{yy}\end{array})(\begin{array}{c}{I}_{x}\\ {I}_{y}\end{array})={T}_{{\rm{lin}}}^{{\rm{f}}}(\begin{array}{c}{I}_{x}\\ {I}_{y}\end{array})$$

If no magneto-optical materials are present, the transmission matrix ${T}_{{\rm{lin}}}^{{\rm{b}}}$ for illumination of the back of the structure is given by the reciprocity theorem^36^,

2 $${T}_{{\rm{lin}}}^{{\rm{b}}}=(\begin{array}{cc}{T}_{xx} & -{T}_{yx}\\ -{T}_{xy} & {T}_{yy}\end{array})$$

We note that illumination of the back of the structure may be achieved either by 180° rotation of the structure around the *y* axis (or any other axis normal to *z*) or by moving the radiation source to the opposite side of the metamaterial. ${T}_{{\rm{lin}}}^{{\rm{b}}}$ characterizes the transmission in the same lab coordinate system as ${T}_{{\rm{lin}}}^{{\rm{f}}}$ with the sample being rotated by 180° with respect to the *y* axis (or *x* axis) and wave propagation along +*z*. (All formulas also apply without sample rotation when describing opposite illumination directions in different coordinate systems defined by wave propagation along +*z*. For forward illumination, these coordinates coincide with the lab coordinates. For backward illumination, 2 axes are inverted: *z* and either *x* or *y*.) Eq. (1) implies that the conversion efficiencies from *x*-to-*y* and *y*-to-*x* polarization may in general be different and Eq. (2) implies that these conversion efficiencies interchange for opposite illumination directions. It follows that the total transmission for a given incident linearly polarized wave may be different for opposite illumination directions. The transmission asymmetry for linearly polarized waves is characterized by

3 $${{\rm{\Delta }}}_{{\rm{lin}}}^{(x)}={|{T}_{yx}|}^{2}-{|{T}_{xy}|}^{2}=-{{\rm{\Delta }}}_{{\rm{lin}}}^{(y)}$$

${{\rm{\Delta }}}_{{\rm{lin}}}^{(x)}$ is the total transmission difference for *x*-polarized waves incident on front and back of the material (front-back). The transmission asymmetry is strongly polarization-dependent, and orthogonal linear polarizations always experience directional transmission asymmetries of same magnitude and opposite sign as indicated by Eq. (3). In the special case where |*T*_*xx*_| = |*T*_*yy*_|, the total transmission difference for *x*-polarized waves incident on front and back of the material is the same as the total transmission difference for *x*- and *y*-polarized waves incident on the front of the material (*x*-*y*). Similarly, the transmission asymmetry for circularly polarized waves is characterized by

4 $${{\rm{\Delta }}}_{{\rm{circ}}}^{(+)}={|{T}_{-+}|}^{2}-{|{T}_{+-}|}^{2}=-{{\rm{\Delta }}}_{{\rm{circ}}}^{(-)}$$

where *T*_−+_ and *T*_+−_ refer to the cross-polarized conversion coefficients of the Jones matrix for right-handed (RCP, ‘+’) and left-handed (LCP, ‘−’) circularly polarized waves illuminating the front of the metamaterial, with detailed form as follows

5 $${T}_{{\rm{circ}}}^{{\rm{f}}}=(\begin{array}{cc}{T}_{++} & {T}_{+-}\\ {T}_{-+} & {T}_{--}\end{array})=\frac{1}{2}(\begin{array}{cc}{T}_{xx}+{T}_{yy}+i({T}_{xy}-{T}_{yx}) & {T}_{xx}-{T}_{yy}-i({T}_{xy}+{T}_{yx})\\ {T}_{xx}-{T}_{yy}+i({T}_{xy}+{T}_{yx}) & {T}_{xx}+{T}_{yy}-i({T}_{xy}-{T}_{yx})\end{array})$$

Here we define RCP as clockwise rotation of the electric field at a fixed position as seen by an observer looking into the THz beam. We note that linear anisotropy with polarization eigenstates *x*, *y* is characterized by *T*_*xx*_ ≠ *T*_*yy*_ and *T*_*xy*_ = *T*_*yx*_ = 0, where linear birefringence corresponds to the phase difference and linear dichroism to the amplitude difference between *T*_*xx*_ and *T*_*yy*_. Similarly, optical activity is characterized by *T*_++_ ≠ *T*_--_, where circular birefringence corresponds to the phase difference and circular dichroism to the amplitude difference between *T*_++_ and *T*_--_.

It is worth emphasizing that the transmission properties (Jones matrix components) of a metamaterial structure are determined by its unit cell as well as the spatial arrangement of unit cells, i.e. the local symmetry of each meta-molecule and the global symmetry of the lattice^16^. Reconfiguration of the effective resonant structure provides an opportunity to change the transmission characteristics by manipulating the symmetry of the metamaterial structure.

In order to understand which symmetries prevent asymmetric transmission of linearly polarized waves, let us first consider the transmission characteristics of a symmetric structure. Figure 2(a) shows the chosen unit cell, where a pair of metallic rings sandwiches a dielectric layer symmetrically. The structure exhibits mirror symmetry with respect to the *y*-*z* (or *x*-*z*) and *x*-*y* planes, as well as fourfold rotational symmetry with respect to the *z* axis. Following^37^, we examine the consequences of each symmetry individually.

**Firstly**, Fig. 2(a) exhibits mirror symmetry with respect to the *x*-*z* plane, thus, polarization conversion from *x* to *y* and *x* to -*y* must be equal, *T*_*yx*_ = −*T*_*yx*_. (Similarly, also *T*_*xy*_ = −*T*_*xy*_.) It follows that the off-diagonal elements of the Jones matrix must be zero when the structure is mirror-symmetric with respect to the *x*-*z* plane (*y*-*z* plane), i.e.

6 $${T}_{{\rm{lin}}}^{{\rm{f}}}=(\begin{array}{cc}{T}_{xx} & 0\\ 0 & {T}_{yy}\end{array})$$

Thus, a structure with a plane of mirror symmetry that contains the illumination direction may exhibit linear anisotropy, but not optical activity and no asymmetric transmission phenomena.

**Secondly**, Fig. 2(a) exhibits mirror symmetry with respect to the *x*-*y* plane. Therefore, 180°-rotation of the structure with respect to the *y* axis (i.e. illumination of the opposite side of the structure) is equivalent to inverting the *x* axis. It follows that *x* to *y* polarization conversion for illumination of the structure’s front must be the same as –*x* to *y* polarization conversion for illumination of the structure’s back. Comparison of Eqs (1) and (2), describing front and back illumination, shows that this implies *T*_*xy*_ = *T*_*yx*_. It follows that the off-diagonal matrix elements must be identical when the structure is mirror-symmetric with respect to the *x*-*y* plane, i.e.

7 $${T}_{{\rm{lin}}}^{{\rm{f}}}=(\begin{array}{cc}{T}_{xx} & {T}_{xy}\\ {T}_{xy} & {T}_{yy}\end{array})$$

Thus, a structure with a plane of mirror symmetry perpendicular to the illumination direction may exhibit linear anisotropy and asymmetric transmission of circularly polarized waves, but not optical activity and asymmetric transmission of linearly polarized waves.

**Thirdly**, Fig. 2(a) exhibits fourfold rotational symmetry with respect to the *z* axis, thus, 90°-rotation of the structure with respect to the *z* axis will not change the experiment, implying identical properties for incident *x* and *y* polarizations, i.e. *T*_*xx*_ = *T*_*yy*_ and *T*_*yx*_ = −*T*_*xy*_. It follows that the diagonal matrix elements must be identical and that one off-diagonal matrix element must be the negative of the other when the structure exhibits fourfold rotational symmetry with respect to the *z* axis, i.e.

8 $${T}_{{\rm{lin}}}^{{\rm{f}}}=(\begin{array}{cc}{T}_{xx} & {T}_{xy}\\ -{T}_{xy} & {T}_{xx}\end{array})$$

Thus, a structure that has fourfold rotational symmetry with respect to the illumination direction may exhibit optical activity, but cannot exhibit any linear anisotropy or asymmetric transmission phenomena for linearly or circularly polarized waves.

For our symmetric structure, Eqs (6–8) apply simultaneously, implying that the symmetric metamaterial design shown in Fig. 2(a) cannot change the polarization state of a normally incident wave upon transmission (*T*_*xx*_ = *T*_*yy*_, *T*_*yx*_ = *T*_*xy*_ = 0). In particular, asymmetric transmission for linearly polarized waves requires the mirror symmetry and fourfold rotational symmetry to be broken simultaneously^17^.

Breaking the symmetry provides additional degrees of freedom to obtain engineered functionality that enables the control of polarization states. If the metallic rings in Fig. 2(a) are cut to realize a pair of mutually twisted SRRs, as shown in Fig. 2(b,c), we get an asymmetric structure that lacks both mirror symmetry and fourfold rotational symmetry^19,20^. Such a structure, with a mutual twist of 90°, can be analyzed from Fig. 2(d). The structure has twofold rotational symmetry with respect to the *y*′ direction. The coordinate system (*x*′, *y*′, *z*′) has been rotated by *θ* = 45° with respect the (*x*, *y*, *z*) coordinates about the *z* axis. Experiments where a *y*′-polarized (or *x*′-polarized) wave illuminates either front or back of the slab are indistinguishable^20^ and therefore, ${T}_{{\rm{lin}}}^{{\rm{f}}}$ and ${T}_{{\rm{lin}}}^{{\rm{b}}}$ must be identical in the (*x*′, *y*′, *z*′) coordinates. Considering Eqs (1) and (2) for these coordinates, we have

9 $${T}_{x^{\prime} y^{\prime} }=-{T}_{y^{\prime} x^{\prime} }$$

implying absence of asymmetric transmission for *x’* and *y’*-polarized waves. The transmission matrix in the (*x*′, *y*′, *z*′) coordinates can be obtained through coordinate transformation from the *T* matrix in the (*x*, *y*, *z*) coordinates, which can be expressed as

10 $$(\begin{array}{cc}{T}_{x^{\prime} x^{\prime} } & {T}_{x^{\prime} y^{\prime} }\\ {T}_{y^{\prime} x^{\prime} } & {T}_{y^{\prime} y^{\prime} }\end{array})=J(\begin{array}{cc}{T}_{xx} & {T}_{xy}\\ {T}_{yx} & {T}_{yy}\end{array}){J}^{-1}$$

where *J* is the Jacobi matrix for the coordinate rotation

11 $$J=(\begin{array}{cc}\cos \,\theta  & \sin \,\theta \\ -\sin \,\theta  & \cos \,\theta \end{array})=\frac{1}{\sqrt{2}}(\begin{array}{cc}1 & 1\\ -1 & 1\end{array})$$

Substituting Eqs (9) and (11) into (10), the following relation was obtained^20^

12 $${T}_{xx}={T}_{yy}$$

It follows from Eqs (4) and (5) that the asymmetric transmission parameter for circularly polarized waves must be zero. Thus, the conditions for asymmetric transmission of linear polarization, but not circular polarization, are fulfilled by this chiral split ring metamaterial. In order to observe dynamic switching of polarization conversion and asymmetric transmission, an active material can be employed to modify the effective resonant structure. VO_2_ is a suitable material that exhibits an insulator to metal phase transition under electric, thermal, and optical excitation, resulting in a more than four orders of magnitude change of electrical conductivity.

## Results and Discussion

**Figure 3:**
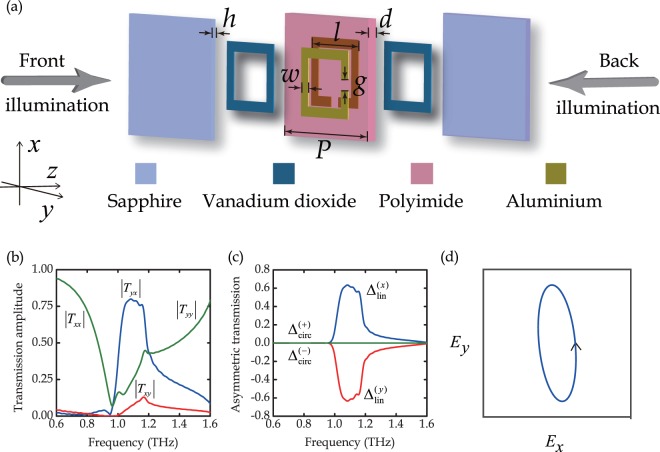
Metamaterial structure and simulated electromagnetic properties at room temperature. (**a**) Schematic of the unit cell exhibiting asymmetric transmission of linearly polarized waves, with parameters *g* = 5 μm, *w* = 8 μm, *l* = 49 μm, *d* = 11 μm, *h* = 500 μm and *P* = 82 μm. (**b**) Co- and cross-polarized spectral response |*T*_*xx*_|, |*T*_*yx*_|, |*T*_*xy*_|, and |*T*_*yy*_| of the metamaterial, where *T*_*ij*_ represents the transmission coefficient describing *i*-polarized transmitted waves resulting from *j*-polarized illumination of the metamaterial’s front. (**c**) Asymmetric transmission for linearly and circularly polarized incident waves, where ‘−’ and ‘+’ correspond to left- and right-handed circularly polarized waves. (**d**) Polarization ellipse of the transmitted wave at 1.1 THz for *x*-polarized illumination of the metamaterial’s front.

Based on the above analysis, we chose the asymmetric metamaterial unit cell shown in Fig. 3(a): a 90°-twisted metallic SRR dimer made of 200 nm-thick aluminum layers separated by a polyimide spacer. The SRRs were covered by 150 nm-thick VO_2_ square rings, and two sapphire substrates support the VO_2_ films and metallic SRRs. Guided by numerical simulations, the metamaterial was fabricated by conventional photolithography and its transmission and polarization properties were then characterized by THz time-domain spectroscopy as explained in the Methods.

Figure 3(b) shows the metamaterial’s simulated transmission characteristics at room temperature, when VO_2_ is in its insulator phase. |*T*_*xx*_| and |*T*_*yy*_| are equal throughout the simulated frequency range and exhibit a broad resonant dip at 0.96 THz. The cross-polarized transmission amplitude |*T*_*yx*_| peaks at 0.8 around 1.1 THz, while |*T*_*xy*_| remains below 0.13 in the entire 0.6–1.6 THz frequency range. The difference between the cross-polarized transmission amplitudes results in a directional transmission asymmetry for linearly polarized waves, ${{\rm{\Delta }}}_{{\rm{lin}}}^{(x)}={|{T}_{yx}|}^{2}-{|{T}_{xy}|}^{2}$, that reaches a very large value of 0.62 at the resonance frequency of 1.1 THz, see Fig. 3(c). Thus, at the 1.1 THz resonance, our simulations indicate that the structure is 62% more transparent for *x*-polarized waves incident on its front than its back in terms of intensity. The corresponding asymmetry for the opposite polarization has the same magnitude and the opposite sign, ${{\rm{\Delta }}}_{{\rm{lin}}}^{(y)}=-\,{{\rm{\Delta }}}_{{\rm{lin}}}^{(x)}$. Our simulations confirm further that asymmetric transmission for circularly polarized waves is absent, ${{\rm{\Delta }}}_{{\rm{circ}}}^{(+)}=0$ and ${{\rm{\Delta }}}_{{\rm{circ}}}^{(-)}=0$, at all frequencies due to the metamaterial’s symmetry as discussed in the theory section and control experiments confirm this within experimental accuracy. Furthermore, the chiral and anisotropic metamaterial can cause dramatic polarization changes. Figure 3(d) shows the polarization state of the transmitted wave for illumination of the metamaterial’s front with an *x*-polarized, 1.1 THz wave. In this case, the polarization azimuth of the transmitted wave is rotated by −84° and the transmitted wave acquires an ellipticity angle of −17°, i.e. the *x*-polarized incident wave is approximately converted to the orthogonal *y*-polarized wave.

**Figure 4:**
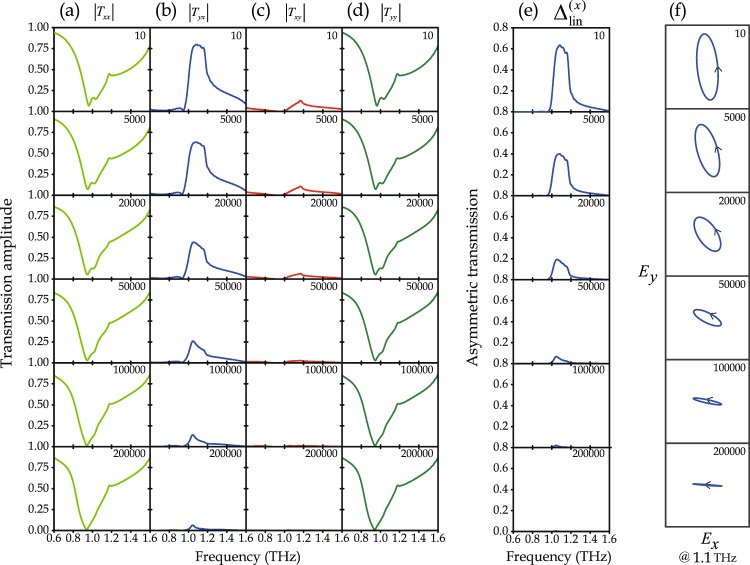
Simulated frequency dependence of co- and cross-polarized transmission amplitudes for *x*- and *y*-polarized waves incident on the metamaterial’s front with VO_2_ conductivity ranging from 10 S/m to 200000 S/m. Columns (**a–d**) show |*T*_*xx*_|, |*T*_*yx*_|, |*T*_*xy*_|, and |*T*_*yy*_| respectively. (**e**) Frequency dependence of the asymmetric transmission parameter for linearly polarized waves, and (**f**) transmitted polarization state at 1.1 THz for incident *x*-polarization.

**Figure 5:**
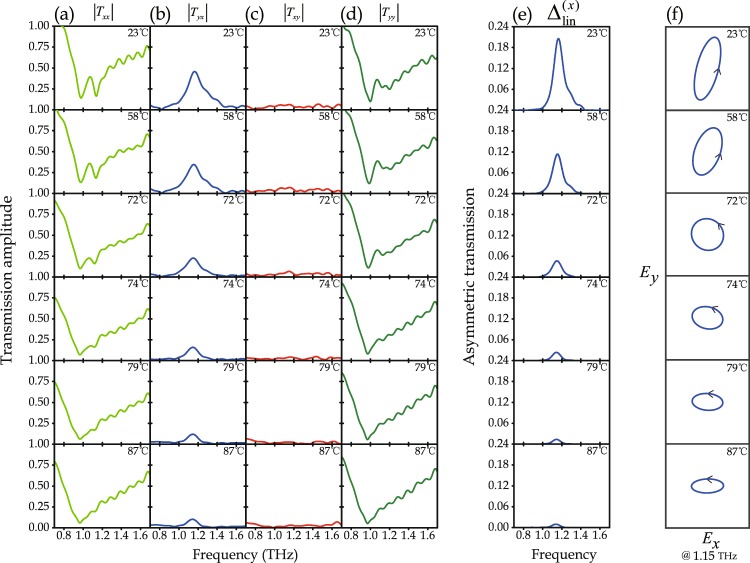
Measured frequency dependence of co- and cross-polarized transmission amplitudes for *x*- and *y*-polarized waves incident on the metamaterial’s front at temperatures ranging from 23 °C to 87 °C. Columns (**a**–**d**) show |*T*_*xx*_|, |*T*_*yx*_|, |*T*_*xy*_|, and |*T*_*yy*_| respectively. (**e**) Frequency dependence of the asymmetric transmission parameter for linearly polarized waves, and (**f**) transmitted polarization state at 1.15 THz for illumination with *x*-polarized waves.

The metamaterial’s transmission characteristics were simulated as a function of VO_2_ conductivity (Fig. 4) and measured as a function of temperature (Fig. 5). We simulated and measured the co- and cross-polarized transmission amplitudes, transmission asymmetry, and transmitted polarization state for linearly polarized waves incident on the metamaterial’s front. At room temperature (23 °C), VO_2_ is in its insulator state described by a conductivity of 10 S/m^30^, corresponding to disconnected SRR gaps. As VO_2_ is heated, it gradually switches to its metallic phase described by a conductivity of 200000 S/m^30,33–35^, corresponding to short-circuited SRR gaps. Therefore, we modelled the metamaterial with VO_2_ conductivities from 10 to 200000 S/m and compare to the measured metamaterial properties in the experimentally accessible temperature range of 23 to 87 °C.

Simulations (Fig. 4(a,d)) and experiments (Fig. 5(a,d)) show that the co-polarized transmission amplitudes, |*T*_*xx*_| and |*T*_*yy*_|, are the same within numerical and experimental accuracy and exhibit a broad resonant minimum just below 1 THz that hardly changes with VO_2_ conductivity or temperature. In contrast, the cross-polarized transmission amplitudes, |*T*_*yx*_| and |*T*_*xy*_|, are different from each other and strongly dependent on VO_2_ conductivity and temperature (Figs 4(b,c) and 5(b,c)). The cross-polarized transmission amplitudes are largest when the conductivity of the VO_2_ rings is low, 10 S/m (low temperature, 23 °C). With increasing conductivity (temperature), the shape of the spectral dependence of cross-polarized transmission remains similar, but its magnitude decreases monotonously and dramatically, vanishing almost completely at a conductivity of 200000 S/m (temperature of 87 °C). |*T*_*yx*_| reaches a large maximum near 1.1 THz (0.8 simulated at 10 S/m, 0.45 measured at 23 °C), while |*T*_*xy*_| remains much smaller throughout the entire 0.6–1.6 THz spectral range (<0.13 simulated, <0.06 measured). It is the difference between |*T*_*yx*_| and |*T*_*xy*_| that leads to asymmetric transmission of linearly polarized waves.

Figures 4(e) and 5(e) show the simulated VO_2_-conductivity-dependence and measured temperature-dependence of the asymmetric transmission parameter ${{\rm{\Delta }}}_{{\rm{lin}}}^{(x)}$, revealing a remarkable switching effect. For the room temperature conductivity of VO_2_ (10 S/m), the simulated ${{\rm{\Delta }}}_{{\rm{lin}}}^{(x)}$ exceeds 0.62 at 1.1 THz, corresponding to a huge transmission asymmetry of more than 62% in terms of intensity for *x*-polarized waves incident on front and back of the structure. A large asymmetry exceeding 20% was detected experimentally at 23 °C at 1.15 THz. Both simulations and experiments show that, with increasing VO_2_ conductivity (temperature), the shape of the transmission asymmetry’s spectral dependence does not change much, but its magnitude reduces continuously and dramatically, vanishing almost completely in the entire 0.6–1.6 THz spectral range at 200000 S/m (87 °C). Thus, we observe temperature-controlled asymmetric transmission of linearly polarized THz waves. The switching effect allows the large transmission asymmetry that occurs at room temperature to be switched off by heating the metamaterial.

In this metamaterial, thermal switching of asymmetric transmission of linearly polarized waves is accompanied by large changes of the transmitted polarization state. This is illustrated for transmission of *x*-polarized waves incident on the metamaterial’s front (Fig. 4(f): simulations at 1.1 THz, Fig. 5(f): measurements at 1.15 THz). At room temperature, when VO_2_ is in its insulating state (10 S/m), the metamaterial converts the incident *x*-polarized waves approximately to *y*-polarized waves. The polarization change becomes smaller with increasing temperature as VO_2_ switches to its metallic state. At a VO_2_ conductivity of 200000 S/m (temperature of 87 °C), the transmitted polarization state remains approximately *x*-polarized. Simulations and experiments show that the polarization azimuth and ellipticity angle of the transmitted polarization state can be controlled by changing the metamaterial’s temperature. The accessible azimuth range is close to 90° in simulations and experiments. The transmitted wave was generally found to have left-handed elliptical polarization, reaching ellipticity angles of −1.5° to −26° in simulations and −23° to −42° in experiments, where ellipticity angles of 0° and −45° correspond to linear and left-handed circular polarization, respectively. Thus, the results show that the metamaterial approximately converts *x*-polarization to *y*-polarization at room temperature and that the polarization change can be controlled and switched off by heating the structure.

While our simulations and experiments show the same qualitative behavior, we note that quantitative differences result from fabrication imperfections. A small shift of asymmetric transmission frequencies and significantly smaller experimental transmission asymmetries are mainly due to SRR fabrication tolerances, thickness errors of the polyimide spacer, small air gaps between SRRs and polyimide spacer and misalignment between front and back SRR layers.

**Figure 6:**
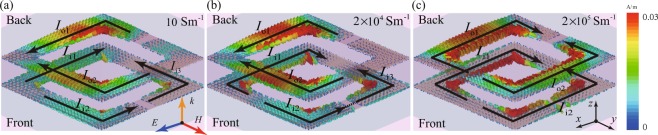
Snapshots of surface currents in a unit cell of the asymmetric transmission metamaterial at 1.1 THz for VO_2_ film conductivities of (**a**) 10 S/m, (**b**) 20000 S/m, and (**c**) 200000 S/m. The incident wave is *x*-polarized and illuminates the front of the structure in all cases.

Figure 6 shows how the surface currents excited by an *x*-polarized wave incident on the metamaterials’s front depend on the conductivity of the VO_2_ rings at 1.1 THz. When VO_2_ is in its insulator state (10 S/m conductivity, Fig. 6(a)), the incident wave excites a resonance characterized by three current oscillations, *I*_i1_, *I*_i2_, and *I*_i3_, separated by current nodes in the input split ring. The parallel conductors of the input and output split rings yield antisymmetric current oscillation pairs (*I*_i1_, *I*_o1_) and (*I*_i2_, *I*_o2_) in input and output split rings, which correspond to magnetic dipoles. No such current oscillation pair can be formed with *I*_i3_, due to the insulating gap in the output split ring. This breaks the symmetry of the metamaterial excitation and results in re-radiation of THz waves with a polarization change. The metamaterial excitation remains qualitatively similar up to moderately large VO_2_ conductivities around 20000 S/m (Fig. 6(b)). But when VO_2_ reaches its metallic state (200000 S/m conductivity, Fig. 6(c)), the SRRs effectively become rings without splits, which can only support symmetric current oscillations. The fundamental symmetric mode, characterized by 2 current oscillations separated by nodes, is excited in the input and output rings. No polarization conversion is possible in the ideal case corresponding to charge oscillations that are perfectly symmetric with respect to the *x*-polarization of the incident wave.

In this work, asymmetric transmission of linearly polarized waves is caused by intrinsic 3D chirality of the anisotropic, lossy metamaterial unit cell when VO_2_ is in its insulating phase. Asymmetric transmission due to intrinsic 3D chirality occurs at normal incidence (as shown by our experiments and simulations). Control simulations at 1.1 THz in the *xz* and *yz* planes of incidence show a minor reduction of the transmission asymmetry from 62% at normal incidence to asymmetries between 56% and 62% for angles of incidence of up to 10°. This weak dependence of our intrinsically 3D-chiral metamaterial’s transmission asymmetry on small variations of the angle of incidence is very different from asymmetric transmission due to extrinsic 3D chirality. Asymmetric transmission of linearly polarized waves due to extrinsic 3D chirality arises from a 3D-chiral experimental arrangement formed by oblique incidence of an electromagnetic wave onto an achiral structure^18^. As asymmetric transmission due to extrinsic chirality is based on chirality associated with an oblique illumination direction, it is inherently strongly dependent on the angle of incidence and vanishes at normal incidence.

## Conclusion

In summary, we have demonstrated temperature-controlled asymmetric transmission of linearly polarized THz waves. By exploiting the insulator-to-metal phase transition of VO_2_, we switch a metamaterial structure between anisotropic 3D-chiral and isotropic achiral configurations, which turn the transmission asymmetry on and off, respectively. The phase transition occurs gradually and is accompanied by large changes of the transmitted polarization state. This allows continuous thermal tuning of the transmission asymmetry and azimuth and ellipticity of the transmitted polarization state. We have analyzed the effect theoretically, characterized it with numerical simulations, observed it experimentally and explained it in terms of surface current distributions. It has potential applications in active polarization control and directionally asymmetric active devices.

## Methods

### Modelling of metamaterial properties

Numerical simulations of the interaction of the metamaterial with THz waves were performed using CST Microwave Studio^TM^. Polyimide was modelled as a lossy dielectric with a dielectric constant of 2.93 + 0.044*i*. Sapphire was modelled as a lossless dielectric with a dielectric constant of 9.6. Aluminum was modelled as a perfect electric conductor with an electric conductivity of 3.56 × 10^7^ S/m. VO_2_ was assumed to be an insulator at room temperature with a conductivity of 10 S/m^30^ and dielectric constant of 9^31^. The high temperature metallic phase of VO_2_ was described by a conductivity of 200000 S/m^30,33–35^. Control experiments on a VO_2_ film of 150 nm thickness at 1.15 THz confirm the conductivity increase by about four orders of magnitude as the thin film is heated from 23 °C to 87 °C. The polarization azimuth rotation angle $\Psi$ and ellipticity angle $\chi$ were calculated from ${\rm{\Psi }}=\frac{1}{2}{\tan }^{-1}\{\frac{2R\,\cos \,(\phi )}{1-{R}^{2}}\}$ and $\chi =\frac{1}{2}{\sin }^{-1}\{\frac{2R\,\sin \,(\phi )}{1+{R}^{2}}\}$, with *R* = $|{T}_{yx}|/|{T}_{xx}|\,$and $\phi =\angle {T}_{yx}-\angle {T}_{xx}$^12^.

### Metamaterial fabrication

The metamaterial was fabricated by conventional photolithography: 150 nm VO_2_ was deposited on two 500-μm-thick C-cut (0001) sapphire substrates; 200-nm-thick aluminium SRRs were molded on the two VO_2_ islands via lithography and lift off technology; a second lithography step was performed to protect the VO_2_ films in the SRR gaps, and then the exposed VO_2_ was etched using CF_4_/O_2_ plasma; finally, the two identical SRRs patterns were placed on opposite sides of a polyimide spacer with 90° mutual rotation.

### Metamaterial characterization

A photoconductive-antenna-based THz time-domain spectroscopy system (THz-TDS) was employed to characterize the transmission and polarization properties of the hybrid metamaterial. The THz beam illuminated the samples normally and four polarizers were employed to select and transform THz polarizations since only horizontally polarized THz waves are efficiently radiated and detected in this THz-TDS. The transmission spectra were obtained by normalizing the frequency-domain information of the sample to that of a reference consisting of a pair of C-cut sapphire substrates separated by a polyimide spacer. Experimental and simulated polarization states were extracted using the same formulas, which are given in the modelling section above. The temperature of the metamaterial was controlled using a hot plate with a hole for THz wave transmission and the actual sample temperature was measured with a thermometer.

## Data Availability

The data for this paper can be obtained from the University of Southampton ePrints research repository: 10.5258/SOTON/D0634.
